# A165 MALIGNANT TUMORS OF THE SMALL BOWEL DIAGNOSED BY DOUBLE BALLOON ENTEROSCOPY: A FIFTEEN-YEAR EXPERIENCE OF A MEDICAL CENTER IN MID-TAIWAN

**DOI:** 10.1093/jcag/gwad061.165

**Published:** 2024-02-14

**Authors:** Y Wu, J Chou

**Affiliations:** China Medical University Hospital, Taichung, Taiwan; China Medical University Hospital, Taichung, Taiwan

## Abstract

**Background:**

The malignant tumors of the small bowel are relatively rare, and their pre-operative diagnosis is usually difficult

**Aims:**

This study was aimed to investigate the clinical characteristics of patients with malignant small bowel tumors who underwent double balloon enteroscopy. Secondary end points were to evaluate the usefulness and safety of double balloon enteroscopy for the diagnosis of patients with suspected small bowel tumors derived from other previous procedures.

**Methods:**

From January 2008 to June 2023, we retrospectively analyzed consecutive patients who underwent double balloon enteroscopy at a medical center in mid-Taiwan over a 15-year period (Figure 1).

**Results:**

Double balloon enteroscopy were performed in 1001 patients. Small bowel tumors were diagnosed in 141 patients (141/1001; 14.1%), of which 76 patients (53.9%) (43 males, a mean age of 61.89 years) had malignant tumors : 37 had gastrointestinal stromal tumors (49.4%), 16 had adenocarcinoma (20.8%), 10 had metastatic cancer (13.1%), 7 had lymphoma (9.2%), 3 had angiosarcoma (3.9%), 2 had carcinoid (2.6%), and 1 had desmoid tumor (1.3%) (Figure 2).

The indications for double-balloon enteroscopy in patients with malignant small bowel tumors were obscure gastrointestinal bleeding (68.4%). The concordance rate of diagnoses based on double balloon enteroscopy with diagnoses based on small bowel barium study, computed tomography, and capsule endoscopy among patients with small bowel tumors was 54.1%, 65.9% , and 76.9%, respectively. (Figure 3, 4) .

Therapeutic plans were changed to surgery in 67.1 % of patients with malignant small bowel tumors.

Additionally, treatment was added on chemotherapy in 25.6% of patients with malignant small bowel tumors after the results of double-balloon enteroscopy.

**Conclusions:**

Approximately 14.1% of patients who underwent double balloon enteroscopy had small bowel tumors, 53.9% of small bowel tumors are malignant. The most common indication for double balloon enteroscopy in patients with malignant small bowel tumors was obscure gastrointestinal bleeding. Double balloon enteroscopy is a very useful modality in diagnosing malignant small bowel tumors and has an important impact on therapeutic plans and clinical results.

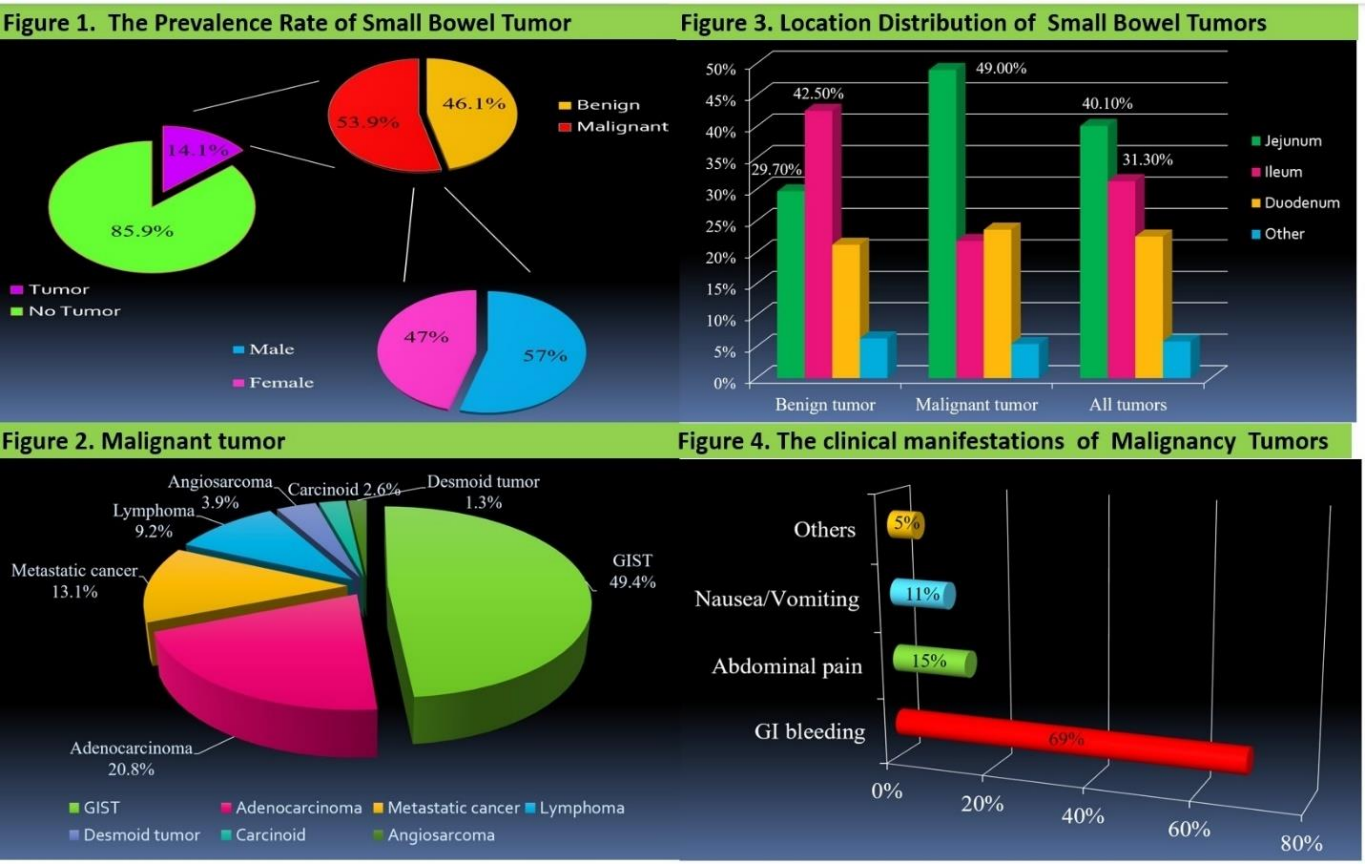

Figure 1. The Prevalence Rate of Small Bowel Tumor

Figure 2. Malignant tumor

Figure 3. Location Distribution of Small Bowel Tumors

Figure 4. The clinical manifestations of Malignancy Tumors

**Funding Agencies:**

None

Intestinal Disorders

